# Nanoparticle Delivery of Immunostimulatory Alu RNA for Cancer Immunotherapy

**DOI:** 10.1158/2767-9764.CRC-22-0354

**Published:** 2023-09-08

**Authors:** Kyle M. Garland, Alexander J. Kwiatkowski, John T. Tossberg, Philip S. Crooke, Thomas M. Aune, John T. Wilson

**Affiliations:** 1 Department of Chemical and Biomolecular Engineering, Vanderbilt University, Nashville, Tennessee.; 2 Department of Medicine, Vanderbilt University Medical Center, Nashville, Tennessee.; 3 Department of Mathematics, Vanderbilt University, Nashville, Tennessee.; 4 Department of Pathology, Microbiology, and Immunology, Vanderbilt University Medical Center, Nashville, Tennessee.; 5 Department of Biomedical Engineering, Vanderbilt University, Nashville, Tennessee.; 6 Vanderbilt Institute for Infection, Immunology, and Inflammation, Vanderbilt University Medical Center, Nashville, Tennessee.; 7 Vanderbilt Center for Immunobiology, Vanderbilt University Medical Center, Nashville, Tennessee.; 8 Vanderbilt-Ingram Cancer Center, Vanderbilt University Medical Center, Nashville, Tennessee.; 9 Vanderbilt Institute of Chemical Biology, Vanderbilt University Medical Center, Nashville, Tennessee.

## Abstract

It was recently found that patients with relapsing remitting multiple sclerosis exhibit widespread loss of adenosine-to-inosine (A-to-I) RNA editing, which contributes to the accumulation of immunostimulatory double-stranded Alu RNA in circulating leukocytes and an attendant increase in levels of proinflammatory cytokines (e.g., type I IFNs). A specific Alu RNA (i.e., AluJb RNA) was implicated in activating multiple RNA-sensing pathways and found to be a potent innate immune agonist. Here, we have performed a bioinformatic analysis of A-to-I RNA editing in human melanoma samples and determined that pre-therapy levels of A-to-I RNA editing negatively correlate with survival times, suggesting that an accumulation of endogenous double-stranded Alu RNA might contribute to cancer patient survival. Furthermore, we demonstrated that immunostimulatory Alu RNA can be leveraged pharmacologically for cancer immunotherapy. AluJb RNA was *in vitro* transcribed and then formulated with endosome-destabilizing polymer nanoparticles to improve intracellular delivery of the RNA and enable activation of RNA-sensing pathways. AluJb RNA/polymer complexes (i.e., Alu-NPs) were engineered to form colloidally stable nanoparticles that exhibited immunostimulatory activity *in vitro* and *in vivo*. Finally, the therapeutic potential of Alu-NPs for the treatment of cancer was demonstrated by attenuated tumor growth and prolonged survival in the B16.F10 murine melanoma tumor model. Thus, these data collectively implicate intratumoral Alu RNA as a potentiator of antitumor innate immunity and identify AluJb RNA as a novel nucleic acid immunotherapeutic for cancer.

**Significance:**

Loss of A-to-I editing leads to accumulation of unedited Alu RNAs that activate innate immunity via RNA-sensing pattern recognition receptors. When packaged into endosome-releasing polymer nanoparticles, AluJB RNA becomes highly immunostimulatory and can be used pharmacologically to inhibit tumor growth in mouse melanoma models. These findings identify Alu RNAs as a new class of nucleic acid innate immune agonists for cancer immunotherapy.

## Introduction

RNA sensing is a prominent defense mechanism employed by mammalian cells to detect intracellular pathogens and cellular distress (1, 2). The abnormal accumulation of RNA at certain locations in or around a cell is a distinguishing feature of many forms of cell stress (e.g., microbial infection, cellular malfunction, etc.; ref. 3). Accordingly, mammalian cells have evolved to express a class of pattern recognition receptors (PRR) known as RNA-sensing PRRs, which can detect (i.e., bind) and respond to such irregularities by activating innate immunity (4–6). Notably, each RNA-sensing PRR is associated with its own recognition requirements (e.g., RNA sequence, RNA morphology, RNA localization, etc.) and distinctive downstream effects (i.e., gene expression signature), which can also depend on cellular and environmental context (7). Once bound to RNA, the RNA-sensing PRRs trigger intracellular signaling cascades that direct the gene expression of the affected cell and ultimately shape an immune response in a stimulus-specific manner. Thus, PRRs have great potential as therapeutic targets for combating a large variety of diseases, because they can be pharmacologically manipulated to orchestrate host immune responses (8).

Under homeostatic conditions, cellular RNAs tend to be immunologically inert and avoid spurious innate immune activation, because RNA-sensing PRRs can largely discriminate against them based on their subcellular localization, composition, and local concentration. Furthermore, cellular RNAs are naturally processed in a variety of ways to avoid innate immune recognition [e.g., 5′ capping, adenosine-to-inosine (A-to-I) editing, etc.]. However, in certain diseases and pathologic states, mechanisms of RNA processing are impaired, resulting in the activation of RNA-sensing PRRs in the absence of pathogen invasion (i.e., sterile inflammation; ref. 9). Indeed, we have recently discovered that circulating leukocytes of patients with relapsing remitting multiple sclerosis (RRMS) exhibit elevated type-I IFN (IFN-I) levels accompanied by an increased presence of double-stranded Alu RNA derived from endogenous Alu elements in the human genome (10). We also found that, while inactive in an edited single-stranded form, certain Alu RNAs can be highly immunostimulatory in an unedited double-stranded form. We attribute the observed increase in double-stranded Alu RNA to a loss of A-to-I editing, because such posttranscriptional modification can destabilize double-stranded RNA (dsRNA) structures (11). In support of this idea, we found in a subsequent study that Alu RNAs in patients with RRMS exhibit widespread loss of A-to-I editing (12). Furthermore, using RNA sequencing (RNA-seq) analysis, we identified several highly expressed Alu RNAs with elevated levels of A-to-I editing in leukocytes from healthy patients relative to those isolated from patients with RRMS.

Of those endogenous Alu RNAs associated with RRMS, one particular dsRNA of the AluJb class (chr17: 76,418,582 – 76,418,856 on the GrCh37/hg19 assembly) was found to be highly immunostimulatory as determined in a reporter cell screen for both IFN-I and NFκB activity (12). Using various reporter cell knockouts, we also determined that the immunostimulatory capacity of the AluJb RNA is primarily derived from its ability to activate both RIG-I and TLR3 (i.e., RNA-sensing PRRs). Notably, our prior results suggest that MDA5 (i.e., another RNA-sensing PRR) is not involved in the recognition of the AluJb RNA, despite the established involvement of this PRR in the detection of other Alu RNAs. The lack of a response from MDA5 might be attributable to the length of the AluJb RNA (i.e., 274 nt), because longer dsRNA molecules (i.e., >300 bp) are required for robust activation of MDA5 (2).

The cytokine signatures associated with both RIG-I and TLR3 activation typically involve type I IFNs and other proinflammatory cytokines that can exert anticancer effects (13–15). Accordingly, localized inflammation induced by administration of Alu RNA as a pharmacologic agent could be beneficial for the treatment of cancer in the context of promoting the cancer-immunity-cycle [i.e., the process through which the immune system can recognize and kill cancer cells (16)]. Indeed, there are several immunostimulatory RNA therapeutics that activate either the RIG-I or TLR3 pathways now in preclinical and/or clinical development for the treatment of solid tumor cancers (17–20). Notably, we have previously demonstrated that AluJb RNA is more potent *in vitro* than poly(IC) RNA in terms of both IFN-I and NFκB activity (12), which suggests that the combined activation of RIG-I and TLR3 has potential to enable heightened innate immune activation relative to the discrete activation of either PRR.

**FIGURE 1:**
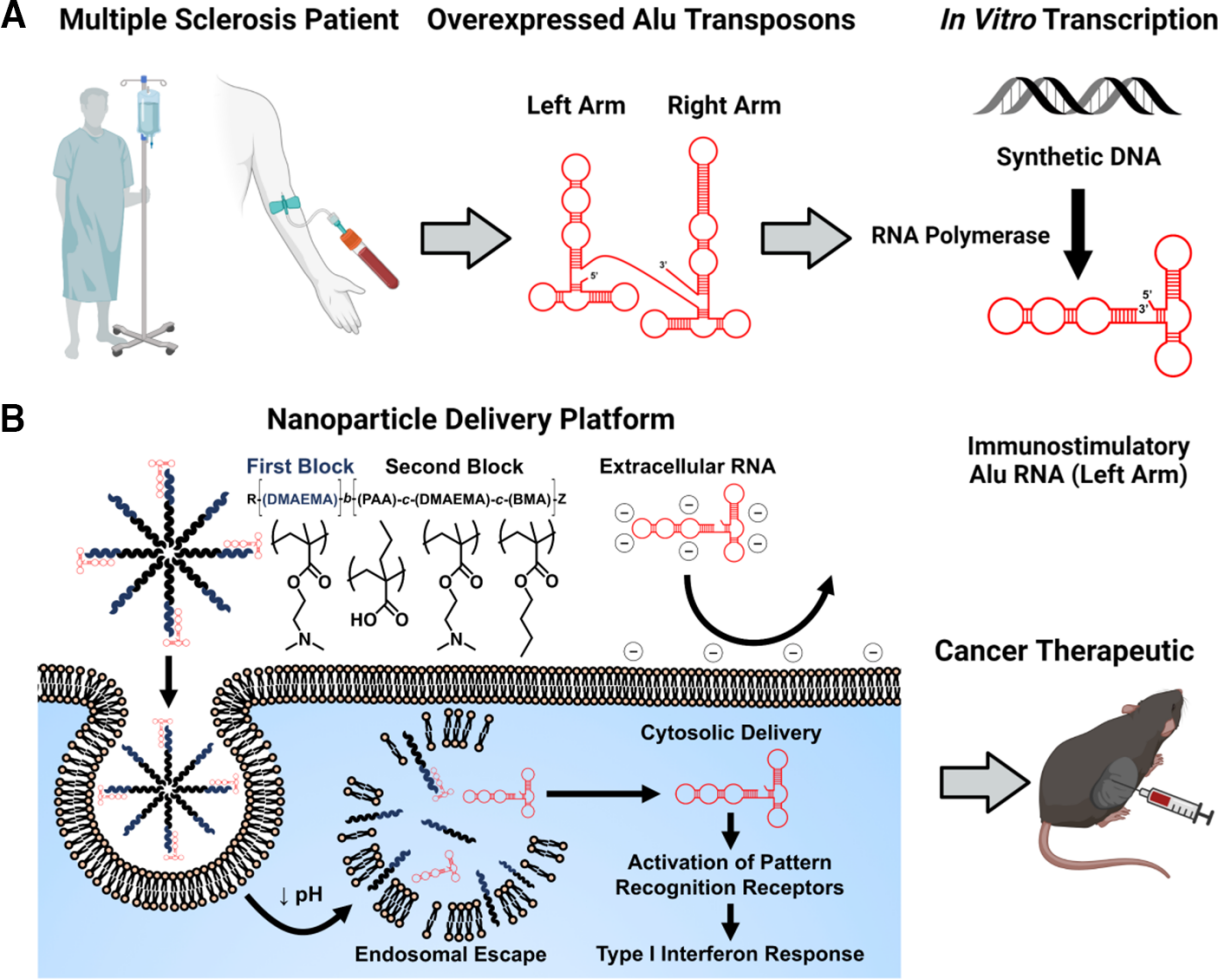
Harnessing Alu RNA for cancer immunotherapy. **A,** Immunostimulatory Alu RNA has been identified in circulating leukocytes of patients with RRMS. *In vitro* transcription of synthetic Alu element DNA can reproduce the immunostimulatory left arm of the Alu RNA. **B,** The immunostimulatory Alu RNA (Left Arm) can be loaded onto an endosomolytic polymer nanoparticle (D-PDB) to yield Alu-NPs that enable cytosolic delivery and activation of RNA-sensing pathways. Utilizing Alu-NPs to activate innate immunity represents a promising strategy to promote antitumor immunity. Figure created with biorender.com.

Here, we have performed a bioinformatic analysis of A-to-I RNA editing in human melanoma samples and determined that pre-therapy levels of A-to-I RNA editing negatively correlate with survival times within certain cancer patient populations. Loss of A-to-I RNA editing, for example by mutations in *ADAR*, results in accumulation of Alu dsRNAs that activate a strong innate immune response (3, 4, 9). Therefore, low levels of A-to-I editing in melanoma may also result in accumulation of endogenous double-stranded Alu RNA that might contribute to patient survival. Furthermore, we have “repurposed” an Alu RNA sequence derived from patients with RRMS for the treatment of cancer by engineering a nucleic acid immunotherapeutic, AluJb RNA (Left Arm), which can efficiently target RNA-sensing PRRs and exploit RNA sensing in the context of local cancer immunotherapy (Fig. 1). To enhance intracellular uptake and cytosolic delivery of noncoding, double-stranded, immunostimulatory Alu RNA, AluJb RNA was electrostatically complexed with a polymeric nanocarrier, poly[(DMAEMA)-*block*-(PAA-*co*-DMAEMA-*co*-BMA)] (D-PDB), previously described by our group and others (18, 21–25). Optimized AluJb RNA (Left Arm)/D-PDB nanoparticles (herein referred to as Alu-NPs) were characterized for nanoparticle stability, *in vitro* transfection efficiency, immunostimulatory activity, and antitumor efficacy. Notably, intratumoral administration of the Alu-NP formulation into murine tumors triggered the expression of proinflammatory cytokines that are known to enhance the tumor infiltration of cancer-killing natural killer cells and T lymphocytes. Finally, therapeutic efficacy of Alu-NP was demonstrated in the B16.F10 murine melanoma tumor model by attenuated tumor growth and prolonged survival. These studies provide proof-of-concept that Alu RNA, here designed to mimic those derived from patients with RRMS, can be leveraged as an innate immune agonist for cancer immunotherapy and establish the Alu-NP platform as a potential new class of RNA therapeutic.

## Materials and Methods

### Bioinformatic Analysis of A-to-I RNA Editing in Human Melanoma Samples

We obtained whole-genome RNA-seq FASTQ files from the National Center for Biotechnology Information's Gene Expression Omnibus to analyze A-to-I RNA editing in patient melanoma samples. We employed the following workflow to identify A-to-I RNA editing sites from paired FASTQ sequencing files. The main identification tool was a python-based package named the SPRINT toolkit that accepts sequence files and produces text files with the following information for each edit site: (i) genomic location, chromosome, and nucleotides from the p terminus, (ii) type of edit; either A-to-G or T-to-C, strand (+ or −), and (iii) number of edits per site and total number of reads per nucleotide site. Mathematica programs were developed to synthesize data including determination of numbers of editing sites within groups unique or shared by groups, mean numbers of total reads and edits for each editing sites, and mean numbers of total reads and edits per total reads across individual gene regions. This information was tied to an Alu database to annotate each A-to-I editing site: location relative to annotated genes and if intronic, noncoding or RNA gene, 3′ or 5′ untranslated regions, or intergenic and if sites were within Alu or non-Alu elements. For these analyses, we defined three categories of A-to-I editing sites: (i) sites with greater than or equal to 1 edit/per read and greater than or equal to 1 read, “all”, (ii) sites with edit/read ratios greater than or equal to 0.05 and greater than or equal to 5 total reads, and (iii) sites with edit/read ratios greater than or equal to 0.25 and greater than or equal to 10 total reads. To create genome-wide A-to-I–editing indices, we identified all A-to-I–editing sites present in one sample and summed edit/read ratios for all editing sites across the genome.

### Materials

D-PDB and DB were synthesized via reversible addition-fragmentation chain transfer polymerization as described previously (22). Alu DNA sequences were obtained from the GrCh37 (hg19) assembly. Synthetic Alu DNA with a SP6 promoter incorporated at the 5′ end was obtained from Integrated DNA Technologies (IDT DNA). Alu RNA was reverse transcribed using synthetic Alu DNA templates (IDT DNA) and MEGAscript SP6 (Invitrogen) in overnight reactions at 37°C. Reaction products were treated with Turbo DNase, precipitated with lithium chloride, and purified using the RNeasy MiniElute Cleanup Kit (Qiagen). Alu RNAs were not treated with phosphatases to remove 5′ phosphate groups. To quantitate yields, absorbance was determined at 260 nm. To ensure that the Alu RNAs were of the predicted size, gel electrophoresis was employed.

### Nanoparticle Formulation

Nanoparticle formulation was performed as described previously (25). Specifically, lyophilized D-PDB or DB polymers were dissolved in ethanol to a concentration of 50 mg/mL. The polymer stock solution was subsequently diluted in phosphate buffer (pH 7.0, 100 mmol/L) to 10 mg/mL, thereby allowing the polymer chains to self-assemble into micelles. Next, four cycles of centrifugal filtration with Amicon Ultra 0.5 mL Centrifugal Filter Units (Ultracel – 3K, Regenerated Cellulose 3,000 NMWL; MilliporeSigma) were employed to concentrate the polymeric micelles into PBS (pH 7.4; Gibco). An aliquot of the concentrated polymeric micelle solution was taken to determine the resultant polymer concentration. The polymer concentration was determined via UV-vis spectroscopy (Synergy H1 Multi-Mode Microplate Reader; Biotek) based on absorbance at 310 nm using a 96-well plate (REF 655180; Greiner Bio-One). The polymeric micelle solution was diluted in PBS (pH 7.4; Gibco) to 1 mg/mL and then sterile filtered (WHA67801302; MilliporeSigma). The sterile-filtered polymer stock solution was then added to an aqueous solution of the desired nucleic acid at molar ratios corresponding with the desired nitrogen-to-phosphate (N/P) charge ratio. For charge ratio calculations, poly(DMAEMA) at a pH 7.4 was estimated to exhibit 50% protonation. The polymer and nucleic acid mixture was rapidly mixed by pipetting and incubated for 20 minutes at room temperature to allow for complete electrostatic complexation.

### Nanoparticle Physical Characterization

Nanoparticle physical characterization was conducted as described previously (25). Dynamic light scattering (DLS; Zetasizer Nano ZS; Malvern Panalytical) was employed to calculate the hydrodynamic sizes of the polymeric micelles and RNA/polymer complexes. Sample was run at pH 7.4 and a polymer concentration of 1 mg/mL. For each 2% agarose gel, 3 g of agarose (16500100; Thermo Fisher Scientific) was dissolved in 150 mL of 1x TAE buffer (REF 46010CM; Corning). The mixture was microwaved until the agarose was fully dissolved and then cast into a gel. The TrackIt 100 bp DNA Ladder (catalog no. 10488058; Thermo Fisher Scientific) was used for all gels unless otherwise indicated. Gel electrophoresis was run at 120 V for 45 minutes. Following electrophoresis, the agarose gels were stained with SYBR Safe dye (S33102; Thermo Fisher Scientific) for 30 minutes in the dark and subsequently imaged (Digital ChemiDoc MP system; Bio-Rad). Transmission electron microscopy (TEM) was conducted on an Osris 200 kV microscope (Tecnai). Briefly samples were dropcast and stained with magnesium tungstate (Nanoprobes) and allowed to dry overnight prior to imaging.

### Cell Lines

All of the cell lines used in this work were maintained according to supplier specifications. RAW-Dual cells (InvivoGen) were cultured in DMEM (Gibco) supplemented with 4.5 g/L glucose, 2 mmol/L l-glutamine, 10% heat-inactivated FBS (Gibco), 100 U mL^−1^ penicillin/100 µg mL^−1^ streptomycin (Gibco), and 100 µg/mL Normocin. To maintain selection pressure of the RAW-Dual cells, 200 µg/mL Zeocin was added every other passage. THP1-Dual cells (InvivoGen) were cultured in RPMI1640 Medium (Gibco) supplemented with 25 mmol/L HEPES, 2 mmol/L l-glutamine, 10% heat-inactivated FBS (Gibco), 100 U mL^−1^ penicillin/100 µg mL^−1^ streptomycin (Gibco), and 100 µg/mL Normocin. To maintain selection pressure of the THP1-Dual cells, 10 µg/mL Blasticidin and 100 µg/mL Zeocin were added every other passage. B16.F10 cells (ATCC) and B16.F10 IFN-LUC cells were cultured in DMEM (Gibco) supplemented with 4.5 g/L glucose, 2 mmol/L l-glutamine, 10% heat-inactivated FBS (Gibco), and 100 U mL^−1^ penicillin/100 µg mL^−1^ streptomycin (Gibco). To maintain selection pressure of the B16.F10 IFN-LUC cells, 10 µg/mL puromycin was added every passage. All of the cell lines used in this work were cultured in a humidified environment (37°C; 5% CO_2_) and routinely tested for *Mycoplasma* contamination.

### *In Vitro* Reporter Cell Assays

The RNA/polymer complexes were screened in 96-well plates (REF 655180; Greiner Bio-One). The wells were seeded with 50,000 reporter cells in 100 µL media. Treatments were diluted to 100 µL in PBS and added to the existing 100 µL media when the wells were approximately 80% confluent. Approximately 24 hours after treatment, cell supernatants were evaluated via the Quanti-Luc (InvivoGen) assay, following manufacturer's instructions. A microplate reader (Synergy H1 Multi-Mode Microplate Reader; Biotek) was used to quantify luminescence and/or absorbance. White, opaque-bottom 96-well plates (REF 655073; Greiner Bio-One) were employed for luminescence measurements. For each sample, signal was calculated from three biological replicates, each with three technical replicates. Signal intensity was normalized by subtracting the average value of a negative control group. For presentation purposes, bell-shaped dose–response curves were truncated at their plateau. Curve fitting analysis (GraphPad Prism software) was employed to estimate EC_50_ values for the various treatments.

### *In Vivo* Imaging Experiments

An IVIS Lumina III (PerkinElmer) was employed to evaluate the *in vivo* IFN activity experiment. Approximately 1 × 10^6^ B16.F10 IFN-LUC cells suspended in 100 µL of PBS were inoculated into the rear right flank of 6–8 weeks old C57BL/6 mice (The Jackson Laboratory) via a subcutaneous injection. When tumors reached a volume of approximately 100 mm^3^, mice were given a single 100 µL intratumoral injection of PBS or Alu-NPs [i.e., AluJb RNA (Left Arm)/D-PDB at an N/P ratio of 4] at a 2 µg RNA dose. Bioluminescence was recorded longitudinally (i.e., 0, 6, 12, and 24 hours). At each predetermined timepoint, the mice were given a dorsal subcutaneous injection of 4.5 mg Pierce d-Luciferin (88293; Thermo Fisher Scientific) solublized in 150 µL PBS, and 15 minutes thereafter, a luminescence image was taken. Mice were shaved and anesthetized via isoflurane gas when necessary.

### Quantitative RT-PCR Analysis

Approximately 1 × 10^6^ B16.F10 cells suspended in 100 µL of PBS were inoculated into the rear right flank of 6–8 weeks old C57BL/6 mice (The Jackson Laboratory) via a subcutaneous injection. When tumors reached a volume of approximately 200 mm^3^, mice were given two 100 µL intratumoral injections administered every 3 days with treatments of either PBS or Alu-NPs [i.e., AluJb RNA (Left Arm)/D-PDB at an N/P ratio of 4] at a 2 µg RNA dose. Mice were euthanized 6 hours after the second intratumoral treatment, and tumors were harvested. A TissueLyser II (Qiagen) was employed to homogenize the tumors, and a RNeasy Plus Mini Kit (Qiagen) was used to isolate tumor RNA.

For the qPCR analysis of proinflammatory gene expression, an iScript cDNA synthesis kit (Bio-Rad) was used according to manufacturer's instructions to reverse transcribe 1 µg of tumor RNA. A CFX Connect Real-time System (Bio-Rad) was employed for qPCR on the resultant cDNA, with the threshold cycle number automatically determined by Bio-Rad CFX manager software V.3.0. The TaqMan gene expression kits (Thermo Fisher Scientific) utilized in this work include mouse *Ppib* (Mm00478295_m1), mouse *Ifnb1* (Mm00439552_s1), mouse *Cxcl10* (Mm00445235_ m1), mouse *Il6* (Mm00446190_m1), and mouse *Tnf* (Mm00443258_m1). For each treatment group, at least four biological samples were evaluated with technical duplicates for the each gene. The threshold cycle numbers of each technical duplicate were averaged. Employing the 2^−ddCt^ method of analysis, gene expression was normalized to the house-keeping gene (i.e., *Ppib*) and subsequently normalized to the negative control (i.e., PBS) treatment values.

### *In Vivo* Tumor Therapy Experiments

Approximately 1 × 10^6^ B16.F10 cells suspended in 100 µL of PBS were inoculated into the rear right flank of 6–8 weeks old C57BL/6 mice (The Jackson Laboratory) via a subcutaneous injection. When tumors reached a volume of approximately 50 mm^3^, mice were given three 100 µL intratumoral injections administered every 3 days with treatments of either PBS, D-PDB polymer NPs, Edited Alu-NPs, or Alu-NPs [i.e., AluJb RNA (Left Arm)/D-PDB at an N/P ratio of 4] at a 2 µg RNA dose or polymer equivalent. Edited Alu-NPs utilized an inactive form of AluJB RNA (Left Arm). Tumor volume, total murine mass, and murine well-being were recorded every other day. A maximum tumor volume of approximately 1,500 mm^3^ was the predetermined study endpoint and used as the basis for “survival” analysis. A T-cell depletion study was employed to evaluate the T-cell dependence of treatment efficacy. Mice were inoculated and treated as described above. However, T-cell depletion via intraperitoneal injection of α-CD8 mAb (BE0061; BioXCell) was initiated 24 hours prior to NP treatment. To this end, 100 µg of α-CD8 mAb was injected on days −1, 2, 5, 8, and 11 (Fig. 5F). Tumor volume, total murine mass, and murine well-being were again recorded *qod*.

### Ethics Statement

The Vanderbilt University Institutional Animal Care and Use Committee (IACUC) reviewed and approved all of the animal experiments performed in this work, and all surgical and experimental procedures were performed in accordance with the regulations and guidelines imposed by the Vanderbilt University IACUC. All mice were maintained under pathogen-free conditions at the animal facilities of Vanderbilt University (Nashville, TN).

### Statistical Analysis

For each experiment, statistical significance was determined as indicated in the corresponding figure caption. GraphPad Prism software (Version 7.0c) was employed for all statistical analyses of this work. Plotted values represent experimental means, and error bars represent one SD, with the exception of those in tumor growth plots, which instead represent one SEM for improved visual presentation. ****, *P* < 0.0001; ***, *P* < 0.005; **, *P* < 0.01; *, *P* < 0.05; ns, not significant.

### Data Availability Statement

The data generated in this study are available upon request from the corresponding author.

## Results

### Analysis of genome-wide A-to-I RNA Editing in Melanoma

We employed the SPRINT software package (26) to measure levels of endogenous A-to-I RNA editing in melanoma tumor samples using whole-genome RNA-seq FASTQ files (27). These analyses scan expressed reads across the genome, identify A-to-G or T-to-C mismatches compared with the reference genome (hg19), filter out known SNPs, report number of mismatches, edits, and number of total reads at each site with a mismatch, and calculate proportion of edits to total reads at each edited site. First, we identified all nucleotide sites across the entire expressed genome with greater than 1 edit and calculated edit/read ratios at each site. Second, to guard against sequencing errors, we identified all editing sites with edit/read ratios greater than 0.05 and total reads greater than 5. We determined both total number of edited sites across the expressed genome and created an A-to-I editing index (AEI) by summing edit/read ratios at each edited site across the expressed genome. We performed this analysis for each melanoma sample with available RNA FASTQ files. These included patient samples both before and after onset of therapy. The study group was comprised of patients with advanced melanoma with the goal of studying tumor responses to either anti-PD-1 therapy, or anti-PD-1 therapy combined with anti-CTLA4. Tumor biopsies were obtained both before (pre-therapy) and 2–4 weeks after (on-therapy) onset of therapy (*N* = 23); whole-genome RNA-seq and whole-exome sequencing were performed [European Genome Phenome Archive Study ID: EGAS00001004545 (19)].

### Negative Correlation Between A-to-I RNA Editing and Time to Death

**FIGURE 2:**
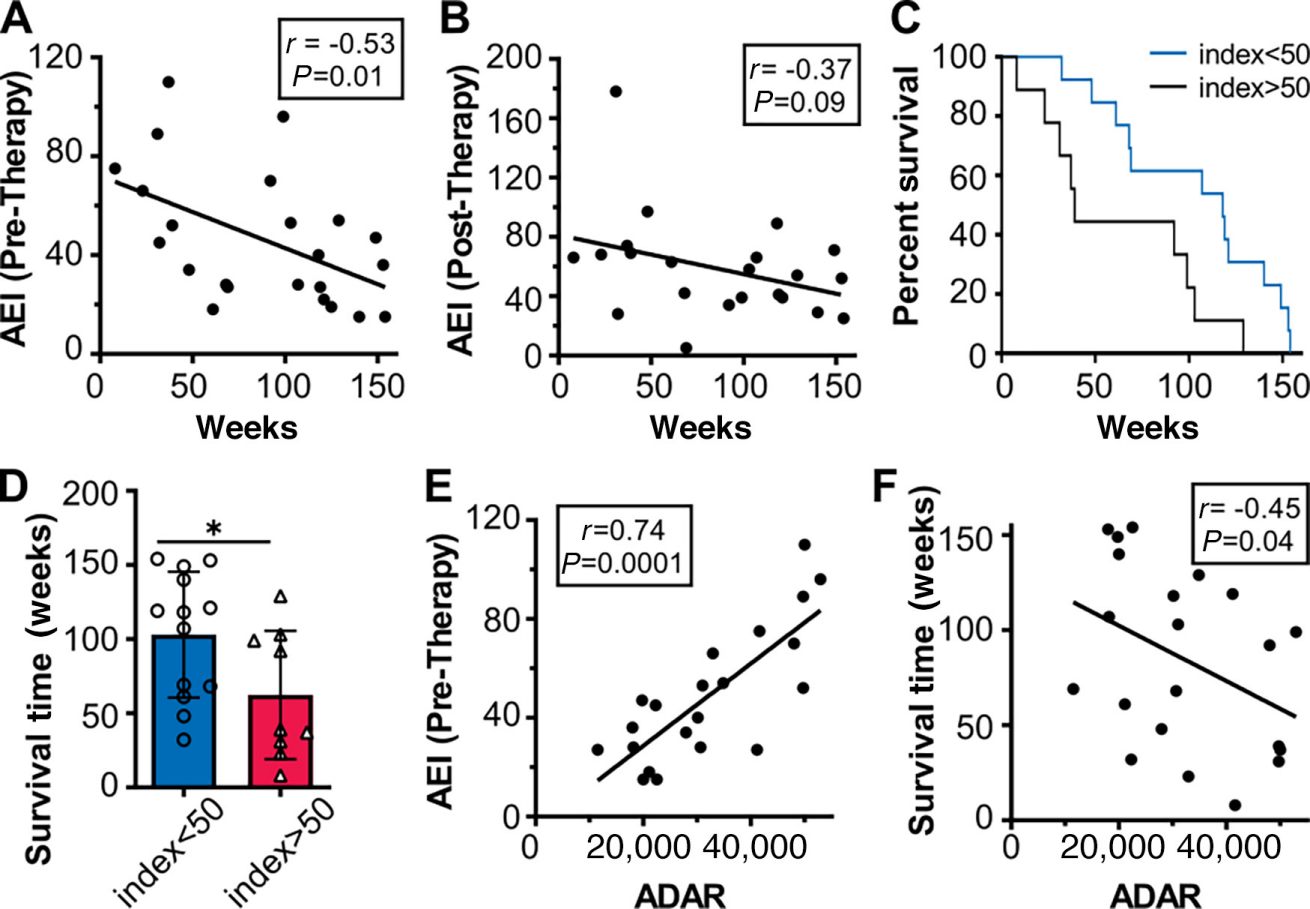
Bioinformatic analysis of A-to-I RNA editing in human melanoma. **A** and **B,** Negative correlation between AEI and time to death. **A,** AEI was determined from RNA-seq data using melanoma samples from patients prior to onset of therapy (*N* = 23). *Y*-axis is AEI for each sample, and *X*-axis is survival time in weeks. Spearman *r* was calculated, and the *P* value was determined by Gaussian approximation. **B,** As in A, except AEI was determined from RNA-seq data using melanoma samples from patients after onset of therapy (*N* = 23). **C,** Kaplan–Meier curve comparing survival time as a function of high (i.e., >50) or low (i.e., <50) pre-therapy AEI. *Y*-axis is percent survival, and *X*-axis is survival time in weeks, *P* = 0.02, log rank (Mantel–Cox test). **D,** As in C, except median survival time was compared in high and low pre-therapy AEI groups, *P* = 0.04; * represents *P* < 0.05. **E** and **F,** Correlation of *ADAR* mRNA levels to pre-therapy AEI and survival. **E,** AEI was determined from RNA-seq data using melanoma samples from patients prior to onset of therapy. *ADAR* mRNA levels for individual melanoma samples were determined from RNA-seq data using the DeSeq package. Spearman *r* was calculated, and the *P* value was determined by Gaussian approximation. **F,** As in E, except *ADAR* mRNA levels are compared with survival times.

We next investigated whether there was a statistically significant correlation between the AEI determined in each melanoma sample both prior and after onset of therapy and survival times (Fig. 2). We found a statistically significant negative correlation between survival times and the AEI determined prior to onset of therapy (Fig. 2A). We also found a negative correlation between the AEI determined after onset of therapy and survival times, but this correlation was not statistically significant (Fig. 2B). We next subdivided melanoma samples from the pre-therapy group into those with high (i.e., >50) or low (i.e., <50) AEIs and performed further analysis by employing the Kaplan–Meier estimator (Fig. 2C). We found statistically significant differences in survival times between samples with low or high AEIs, with the low AEI group exhibiting greater survival times and the high AEI group exhibited reduced survival times. The overall median survival time for the low AEI group was also significantly greater than the overall median survival time for the high AEI group (Fig. 2D). The average AEI was not different between the paired pre-therapy and on-therapy groups (pre-therapy group: average AEI = 48 ± 27, on-therapy group: average AEI = 59 ± 35; unpaired *t* test = 0.25, paired *t* test = 0.14). Overall, we interpret these results to indicate that initial levels of tumor AEI may contribute to overall survival time in melanoma.

The *ADAR* (adenosine deaminase acting on RNA) family of genes encodes for ADAR enzymes that convert adenosine to inosine (28), and so we also determined the correlations between the intratumoral pre-therapy AEI and levels of *ADAR* mRNA and between survival times and levels of *ADAR* mRNA using the DeSeq package. We found a significant positive correlation between ADAR mRNA levels and intratumoral pre-therapy AEI (Fig. 2E). As with the pre-therapy AEI and survival, we found a negative correlation between ADAR mRNA levels and survival time (Fig. 2F). We interpret these results to indicate that expression levels of intratumoral ADAR contribute to the overall intratumoral AEI and that levels of ADAR may be important factors contributing to overall survival time.

### Correlations Between A-to-I RNA Editing Indices and Mutation, Neoantigens, Neopeptides, and Cytolytic Scores

On the basis of previous work by Anagnostou and colleagues investigating correlations between genomic, immunologic, and transcriptomic features and survival in patients with melanoma (27), we evaluated relationships between AEI and several established metrics of tumor immunogenicity. Clinical parameters, including response to therapy, tumor stage and type, and mutation type are summarized (Supplementary Table S1). Major differences between the short and long survival groups were that the short survival groups had more advanced disease, M1C (melanoma has spread to another location that does not involve central nervous system) versus M1A (melanoma has spread only to distant skin and/or soft-tissue sites) and M1B (melanoma has spread to the lung) and greater numbers with progressive disease and failure to respond to therapy. In addition, mutation load, neoantigen load, neopeptide load, and a cytolytic index were determined for each sample. Briefly, mutation load, neoantigen load, and neopeptide loads were determined from whole-exome sequencing data. The cytolytic score was derived from the geometric mean of *GZMA* and *PRF1* mRNA expression levels (19). We compared these additional parameters with the AEI in the pre-therapy group. We did not find a statistically significant correlation between the AEI and mutation load, neoantigen load, neopeptide load, or cytolytic score comparing all samples (Table 1A). We also subdivided samples into low AEI and high AEI and determined whether these two AEI groups exhibited differences in these additional clinical and molecular parameters. Groups with low or high AEI did not exhibit statistically significant differences in mutation load, neoantigen load, neopeptide load, or cytolytic score (Table 1B). Thus, although the AEI is clearly associated with overall survival, the AEI is not associated with these additional metrics of tumor immunogenicity.

**TABLE 1:** Correlation of AEI to mutation, neoantigen, neopeptide, and cytolytic scores

A			B		
Parameter	Spearman *r*	*P*	AEI < 50	AEI > 50	*P*
Mutation load	0.28	ns	234 ± 266	632 ± 948	ns
Neoantigen load	0.17	ns	142 ± 165	363 ± 563	ns
Neopeptide load	0.13	ns	379 ± 525	819 ± 1391	ns
Cytolytic score	0.29	ns	666 ± 557	303 ± 342	ns

NOTE: (**A**) Correlation between indicated parameters and the AEI, Spearman *r*, ns = not significant (# of samples = 22). (**B**) Average ± S.D. of the indicated parameters were determined in samples with AEI less than 50 and AEI greater than 50, ns = not significant.

Multiple sclerosis is associated with loss of A-to-I editing resulting in accumulation of Alu dsRNAs that stimulate IFN regulatory factor and NFkB transcriptional activity and innate immune activation. In melanoma, as shown here, and several other cancers including head and neck, liver, and breast cancer (29), the AEI is associated with survival rates with high AEI associated with poor survival rates compared with cancers with low AEI. We show here that the intratumoral level of ADAR, the gene that encodes adenosine deaminase specific for dsRNA, the major A-to-I editing enzyme, also positively correlates with the AEI and negatively correlates with survival time in melanoma. High AEIs in cancer may result in diversification of the transcriptome via creation of the equivalent of nonsynonymous mutations arising from differential exon editing, splice variants arising from differential editing in introns, and alterations in mRNA stability arising from differential editing in 3′ untranslated regions (30). In addition, high AEI may result in increased editing of Alu dsRNAs preventing their activation of dsRNA sensors and give rise to loss of innate immune homeostasis. This loss of endogenous Alu dsRNAs produced by high levels of A-to-I editing may contribute to reduced survival rates in individuals with advanced melanoma. To begin to explore this latter hypothesis, we evaluated effects of unedited AluJb dsRNA on innate immune activation, tumor growth, and survival in the mouse B16.F10 melanoma model.

### Engineering Alu-NPs to Mimic the Endogenous Expression of Immunostimulatory Alu RNA

The structure-function relationships of the highly immunostimulatory Alu RNA (i.e., AluJb RNA) have recently been reported (12). Because Alu RNAs tend to form two intramolecular double-stranded structures (i.e., left and right arms) separated by an intermediate linker sequence, the importance of each arm in regard to innate immune activation was investigated (12). In our previous studies, each arm was individually *in vitro* transcribed and then screened with the reporter cells for IFN-I and NFκB activity (12). It was determined that the left arm of the AluJb RNA conserved the same immunostimulatory capacity as the full sequence, while the right arm was completely inactive (12). As a result of this finding—and coupled with the fact that shorter nucleic acid sequences are generally easier to load and deliver with nanocarriers—the AluJb RNA (Left Arm) was utilized herein for studies aimed at investigating the therapeutic application of endogenous Alu RNA.

**FIGURE 3:**
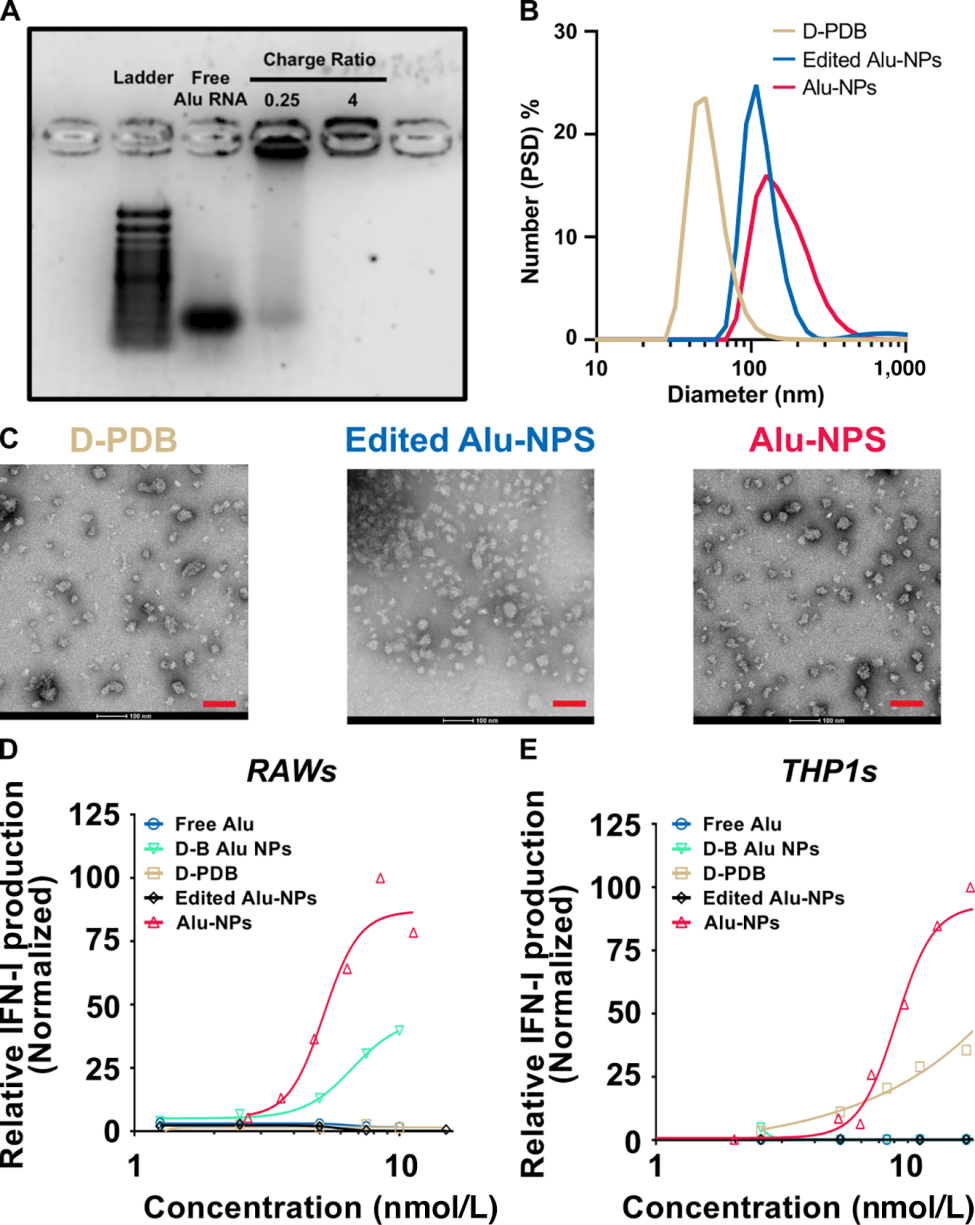
*In vitro* characterization of AluJb RNA (Left Arm)/D-PDB. **A,** Agarose gel of AluJb RNA (Left Arm) with and without various amounts of D-PDB, as indicated. 1 µg RNA/lane. The TrackIt 100 bp DNA Ladder was employed. **B,** DLS analysis of Alu-NPs and Edited Alu-NPs (i.e., AluJb RNA (Left Arm)/D-PDB at an N/P ratio of 4 and Inactive AluJB (Left Arm)/D-PDB at an N/P ratio of 4) relative to D-PDB. **C,** TEM images of D-PDB NPs, Edited Alu-NPs, and Alu-NPs. Scale bars, 100 µm. RAW-Dual (**D**) and THP1-Dual (**E**) reporter cell assays of Alu-NPs, Edited Alu-NPs, D-PDB, D-B Alu NPs, and Free AluJB RNA (Left Arm). All curves are normalized to the maximum value of the Alu-NPs. All experiments were performed at least twice.

To explore whether endogenous Alu RNA could be used to stimulate antitumor immunity, immunostimulatory AluJb RNA (Left Arm) was *in vitro* transcribed, and the endosomolytic polymer, D-PDB was employed to mediate intracellular delivery of the RNA (Fig. 1). While we, and others, have previously leveraged D-PDB for the delivery of nucleic acids, Alu RNAs are unique in their structure and composition, and therefore we first evaluated and optimized the capacity of D-PDB to enhance the activity of AluJb. The synthetic AluJb RNA (Left Arm) was complexed with D-PDB at various N/P charge ratios (i.e., molar amount of protonated amines in the nanoparticle corona/molar amount of phosphates in the nucleic acid) to identify the ratio at which AluJb RNA (Left Arm) is fully loaded onto the polymeric micelles. Gel electrophoresis was performed on the resultant complexes (Fig. 3A), and complete complexation of AluJb RNA (Left Arm) and D-PDB was shown to occur at an N/P charge ratio of 4, which is consistent with previous findings for D-PDB and other nucleic acids (18, 21). At an N/P charge ratio of 4, AluJb RNA (Left Arm)/D-PDB (i.e., Alu-NPs) also demonstrated discrete particle packaging with a uniform particle size distribution centered at approximately 100 nm in diameter (Fig. 3B) with similar particle morphology (Fig. 3C). The lack of particle aggregation also agrees with a previous report (25), which indicates that D-PDB and other similarly sized nucleic acids aggregate only at N/P charge ratios less than 4. Accordingly, an N/P charge ratio of 4 was used for all subsequent experiments involving AluJb RNA (Left Arm)/D-PDB complexes.

The immunostimulatory activity of Alu-NPs was then investigated using *in vitro* activity assays in reporter cell lines. Alu-NP complexes were administered to IFN-I reporter cells that express a secreted luciferase downstream of IFN-stimulated response elements, and 24 hours after treatment, the relative IFN-I production was measured via luminescence (Fig. 3D and E). In both murine RAW-Dual macrophages and human THP1-Dual monocytes, the Alu-NPs demonstrated potent induction of cellular IFN-I production with EC_50_ values of 5.3 nmol/L and 8.3 nmol/L, respectively. These results are in stark contrast to the complete lack of immunostimulatory activity demonstrated *in vitro* by freely administered AluJb RNA (Left Arm) or NPs complexed with edited, inactive AluJB (Left Arm). D-PDB NPs without RNA (i.e.*,* empty NPs) failed to elicit a response in RAW-Dual macrophages and induced only a modest response in THP1-Dual monocytes. Furthermore, while AluJB (Left Arm) complexed with non-endosomolytic DMAEMA-*block*-BMA (D-B) polymer slightly stimulated type-I IFN production in RAW-Dual macrophages, it failed to activate a response in THP1-Dual monocytes. Thus, D-PDB was necessary for effective intracellular delivery of AluJb RNA, and only the unedited form of AluJb RNA was able to potently activate RNA sensing pathways.

### Alu-NPs Stimulate Innate Immunity *In Vivo*

**FIGURE 4:**
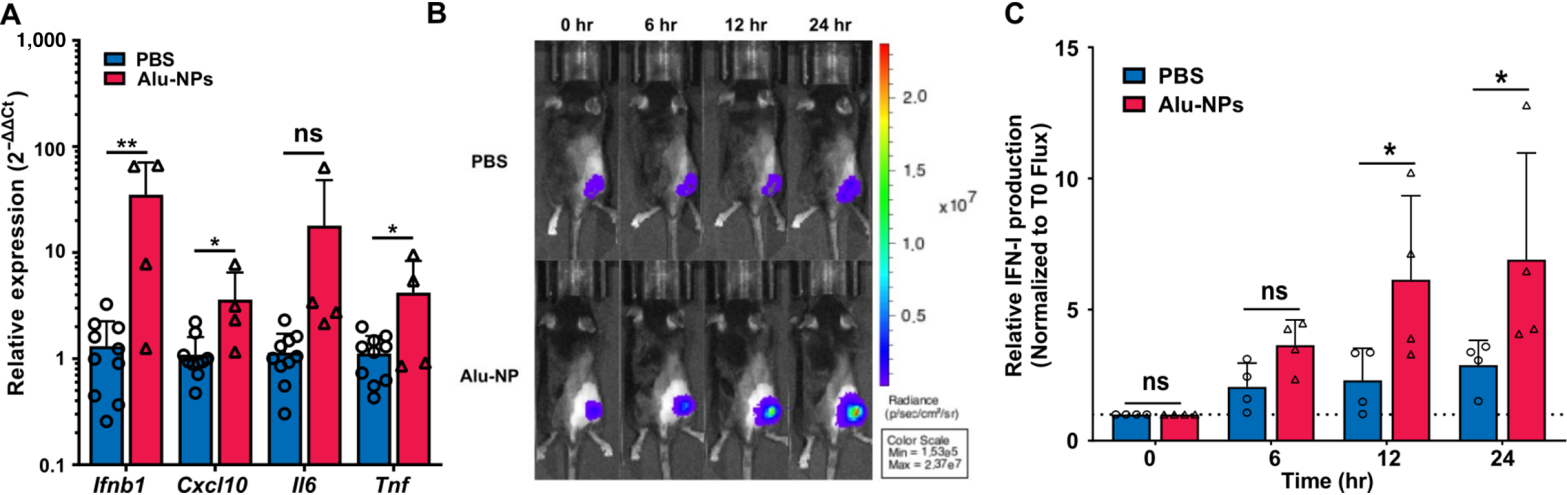
Effects of Alu-NPs on the immune profile of the tumor microenvironment. **A,** qPCR analysis of B16.F10 tumors 6 hours after a second intratumoral injection of PBS or Alu-NPs at a dose corresponding to 2 µg RNA. An unpaired *t* test was used for statistical analysis. ** represents *P* < 0.01 when comparing Alu-NPs with PBS. **B**, Representative IVIS images demonstrating IFN activity in B16.F10 IFN-LUC tumors following a single 100 µL intratumoral injection of either PBS or Alu-NPs at a 2 µg RNA dose. **C,** Quantitative analysis of the *in vivo* IFN activity study. A two-way ANOVA with Sidak test was used for statistical analysis. These experiments were conducted once. * represents *P* < 0.05 when comparing Alu-NPs to PBS.

To determine whether the Alu-NPs conserved their immunostimulatory activity when administered *in vivo*, they were injected intratumorally twice, spaced 3 days apart, in mice bearing subcutaneous B16.F10 tumors, and 6 hours after the second treatment, tumor RNA was collected for qPCR analysis (Fig. 4A). Gene expression changes were quantified for various proinflammatory cytokines that are known to be associated with RIG-I and TLR3 activation (i.e., *Ifnb1*, *Cxcl10*, *Il6*, and *Tnf*). Intratumorally administered Alu-NPs elicited significantly increased gene expression of *Ifnb1*, *Cxcl10*, and *Tnf* in the tumor microenvironment (TME). *Il6* expression also tended to increase relative to PBS-treated tumors, but in a statistically insignificant manner (*P* = 0.086).

Subsequently, B16.F10 IFN-LUC tumors, which express luciferase in response to IFN signaling (31), were used to confirm expression of IFN in the TME and to study the kinetics of signaling in response to intratumorally administered Alu-NPs (Fig. 4B and C). The results demonstrate that Alu-NPs can stimulate significant IFN production within the TME, with peak signal detected at 12–24 hours after treatment. Collectively, these data demonstrate that intratumoral delivery of Alu-NPs could stimulate an innate immune response in the TME.

### Alu-NPs Exert Antitumor Effects in a CD8^+^ T Cell–dependent Manner

**FIGURE 5:**
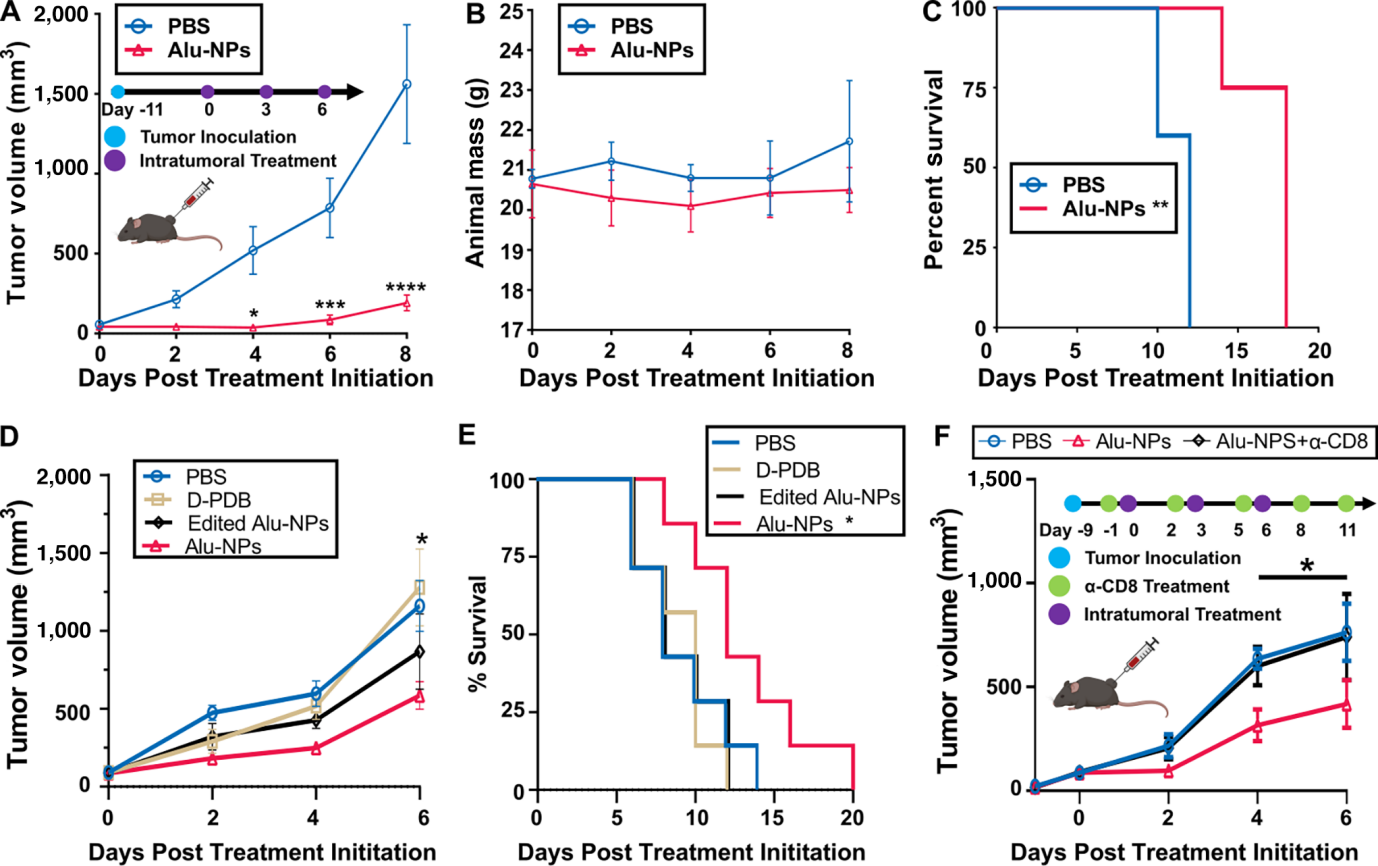
Alu-NPs relay antitumor effects. **A,** Tumor growth curves for B16.F10 tumors treated intratumorally with 100 µL of either PBS or Alu-NPs at a 2 µg RNA dose (*n* = 4 or greater per treatment group). As indicated on the graph, treatments were administered three times every 3 days. The tumor growth curve for each treatment group was truncated to the first day in which a mouse in any treatment group reached the study endpoint. A two-way ANOVA with Sidak test was used for statistical analysis. *, *P* < 0.05; ***, *P* < 0.001; and ****, *P* < 0.0001. **B,** Total mouse weight over time for the mice with B16.F10 tumors. A two-way ANOVA with Sidak test was used for statistical analysis. **C,** Kaplan–Meier survival curve for the mice bearing B16.F10 tumors treated intratumorally with either PBS or Alu-NPs. log-rank (Mantel-C-ox) test was used for statistical analysis. **D,** Tumor growth curves for B16.F10 tumors treated as described in A with empty D-PDB NPs, edited Alu-NPs, or Alu-NPs. Edited Alu-NPs contain Alu RNA that is edited to be inactive (*n* = 7 per group). * represents *P* < 0.05 comparing Alu-NPs to PBS and D-PDB groups. **E,** Kaplan–Meier survival curve of mice treated with empty D-PDB NPs, edited Alu-NPs, or Alu-NPs. **F,** T-cell depletion was initiated with intraperitoneal injection of α-CD8 mAb (100 µg per injection) after tumor inoculation and before treatment. Alu-NPs were statistically different from PBS and Alu-NPs with α-CD8 treatment. Tumor growth curves for B16.F10 tumors after treatment as described in A in mice with or without CD8^+^ T-cell depletion (*n* = 8–9 per group). Experiments in A–E were repeated twice and F was conducted once. * represents *P* < 0.05 comparing Alu-NPs with PBS and Alu-NPs + α-CD8 treated mice. Figure created with biorender.com.

The therapeutic effect of intratumorally administered Alu-NPs was evaluated in the B16.F10 murine melanoma tumor model (Fig. 5). C57BL/6 mice bearing B16.F10 murine melanoma tumors were treated with either vehicle (PBS) or Alu-NPs for a total of three intratumoral injections, each spaced 3 days apart. Relative to PBS treatment, Alu-NP treatment significantly inhibited tumor growth (Fig. 5A), prolonged survival (Fig. 5C), and was well tolerated by the mice as demonstrated by nonsignificant differences in total body mass during or after treatment (Fig. 5B). Importantly, Alu-NPs delayed tumor growth compared with PBS (vehicle) and D-PDB NPs without RNA (Fig. 5D), and neither NPs without RNA nor NPs complexed with an edited inactive Alu RNA conferred the survival benefit of Au-NPs (Fig 5E). To determine whether efficacy was T-cell dependent, CD8^+^ T cells were antibody depleted with the initiation of CD8^+^ T-cell depletion one day prior to the onset of treatment. When Alu-NPs were administered in conjunction with CD8^+^ T-cell depletion, no inhibition of tumor growth was observed, demonstrating that CD8^+^ T cells are required for efficacy (Fig. 5F). These data demonstrate that Alu RNA sequences identified in patients with RRMS can be effectively repurposed for cancer immunotherapy in the form of Alu-NPs.

## Discussion

Our analysis has revealed a negative correlation between endogenous intratumoral RNA AEI and melanoma patient survival. We also find a negative correlation between intratumoral *ADAR* levels, which encodes the major RNA A-to-I editing enzyme, ADAR, and patient survival. These findings are confirmed by comparison of survival times of patients subdivided into high AEI and low AEI groups using Kaplan–Meier survival curves. In contrast, intratumoral AEI does not correlate with other immune parameters including mutation load, neoantigen load, neopeptide load or cytolytic score. The most common A-to-I editing sites are Alu RNAs. Unedited Alu RNAs are potent activators of innate immune responses while edited Alu RNAs are very weak activators of innate immune responses. Increased intratumoral AEI is predicted to result in an increase in levels of edited Alu RNAs compared with unedited Alu RNAs that, in turn, may reduce intratumoral innate immune activation by endogenous immunostimulatory Alu RNAs resulting in decreased survival. To further explore this notion, we tested efficacy of an unedited Alu RNA in the B16.F10 melanoma mouse model.

Double-stranded Alu RNA was successfully “repurposed” as a novel cancer immunotherapy from its role in RRMS-related inflammation. Specifically, immunostimulatory AluJb RNA (Left Arm) was formulated with the endosomolytic polymer, D-PDB to form Alu-NP, which enables the intracellular delivery of RNA and targeted activation of RNA-sensing PRRs. At an N/P charge ratio of 4, Alu-NP complexes exhibited high RNA loading and formed uniform nanoparticles of approximately 100 nm in diameter that could potently activate innate immune signaling pathways as determined with *in vitro* reporter cell assays. As expected, RNA complexation leads to larger NPs diameter than D-PDB alone, but spherical morphology is conserved. Interestingly, D-PDB alone NPs elicited a type I IFN response in THP-1 Dual monocytes, but not RAW-Dual macrophages, while D-B Alu-NPs treatment resulted in the opposite effect. The cell type–specific changes are likely brought on by activation of different PRRs that are preferentially expressed by RAW-Dual Macrophages or THP1-Dual monocytes (32). Upon intratumoral injection, Alu-NPs induced changes in the expression of key immune-related genes, which altered the cytokine profile of the TMEs, resulting in antitumor effects in the B16.F10 murine melanoma tumor model. The combination of *in vitro* (Fig. 3) and *in vivo* (Fig. 5) data supports the conclusion that a carrier delivering active RNA is necessary for efficacy. Notably, free RNA does not stimulate type I IFN production, and polymer alone and Alu-NPs with edited RNA do not provide survival benefit relative to PBS treatment. To the best of our knowledge, this work is the first to demonstrate that immunostimulatory Alu RNAs can be delivered using nanotechnology to stimulate antitumor immune responses.

Regarding future work and applications of Alu-NPs, further characterization of the pharmacokinetic properties of the nanoparticle may be necessary to fully understand the kinetics of signaling and its associated effects on innate immune activation. Moreover, comprehensive cellular and molecular immunophenotyping of the TME in response to Alu-NP remains necessary to establish the major cellular effectors responsible for mediating antitumor responses. However, the lack of antitumor efficacy of Alu-NPs when CD8^+^ T cells are depleted shows that CD8^+^ T cells are necessary and provides a starting point for future mechanistic studies. In addition, other nanocarriers (e.g., lipid nanoparticles) exist and have potential to further enhance delivery of Alu RNA, including advanced polymeric carriers recently described by our group (33, 34), and therefore optimization of Alu-NP formulations with improved properties and enhanced therapeutic efficacy will be important in the translation of Alu RNA for cancer immunotherapy. Finally, the immunostimulatory properties of Alu RNA could also be leveraged for other applications; for example, as a vaccine adjuvant, because vaccines necessitate the use of adjuvants that enable the activation of innate immunity to provide long-lasting protection against pathogenic challenge. Collectively, our studies identify Alu RNA, here designed to mimic that derived from patients with RRMS, as a potent innate immune agonist with potential utility as a cancer immunotherapy.

## Supplementary Material

Supplementary Table 1 Clinical Characteristics from Anagnostou et al.

## References

[1] Hartmann G . Nucleic acid immunity. Adv Immunol 2017;133:121–69. 28215278 10.1016/bs.ai.2016.11.001 PMC7112058

[2] Schlee M , Hartmann G. Discriminating self from non-self in nucleic acid sensing. Nat Rev Immunol 2016;16:566–80. 27455396 10.1038/nri.2016.78 PMC7097691

[3] Roers A , Hiller B, Hornung V. Recognition of endogenous nucleic acids by the innate immune system. Immunity 2016;44:739–54. 27096317 10.1016/j.immuni.2016.04.002

[4] Chen N , Xia P, Li S, Zhang T, Wang TT, Zhu J. RNA sensors of the innate immune system and their detection of pathogens. IUBMB Life 2017;69:297–304. 28374903 10.1002/iub.1625 PMC7165898

[5] Chow KT , Gale M Jr, Loo YM. RIG-I and other RNA sensors in antiviral immunity. Annu Rev Immunol 2018;36:667–94. 29677479 10.1146/annurev-immunol-042617-053309

[6] Uehata T , Takeuchi O. RNA recognition and immunity-innate immune sensing and its posttranscriptional regulation mechanisms. Cells 2020;9:1701. 32708595 10.3390/cells9071701 PMC7407594

[7] Vanpouille-Box C , Hoffmann JA, Galluzzi L. Pharmacological modulation of nucleic acid sensors – therapeutic potential and persisting obstacles. Nat Rev Drug Discov 2019;18:845–67. 31554927 10.1038/s41573-019-0043-2

[8] Mcwhirter SM , Jefferies CA. Nucleic acid sensors as therapeutic targets for human disease. Immunity 2020;53:78–97. 32668230 10.1016/j.immuni.2020.04.004

[9] Rehwinkel J , Gack MU. RIG-I-like receptors: their regulation and roles in RNA sensing. Nat Rev Immunol 2020;20:537–51. 32203325 10.1038/s41577-020-0288-3 PMC7094958

[10] Heinrich MJ , Purcell CA, Pruijssers AJ, Zhao Y, Spurlock CF 3rd, Sriram S, . Endogenous double-stranded Alu RNA elements stimulate IFN-responses in relapsing remitting multiple sclerosis. J Autoimmun 2019;100:40–51. 30826177 10.1016/j.jaut.2019.02.003 PMC6513682

[11] Samuel CE . Adenosine deaminase acting on RNA (ADAR1), a suppressor of double-stranded RNA-triggered innate immune responses. J Biol Chem 2019;294:1710–20. 30710018 10.1074/jbc.TM118.004166 PMC6364763

[12] Tossberg JT , Heinrich RM, Farley VM, Crooke PS 3rd, Aune TM. Adenosine-to-inosine RNA editing of alu double-stranded (ds)RNAs is markedly decreased in multiple sclerosis and unedited Alu dsRNAs are potent activators of proinflammatory transcriptional responses. J Immunol 2020;205:2606–17. 33046502 10.4049/jimmunol.2000384 PMC7872017

[13] Zitvogel L , Galluzzi L, Kepp O, Smyth MJ, Kroemer G. Type I interferons in anticancer immunity. Nat Rev Immunol 2015;15:405–14. 26027717 10.1038/nri3845

[14] Bourquin C , Pommier A, Hotz C. Harnessing the immune system to fight cancer with Toll-like receptor and RIG-I-like receptor agonists. Pharmacol Res 2020;154:104192. 30836160 10.1016/j.phrs.2019.03.001

[15] Elion DL , Cook RS. Harnessing RIG-I and intrinsic immunity in the tumor microenvironment for therapeutic cancer treatment. Oncotarget 2018;9:29007–17. 29989043 10.18632/oncotarget.25626 PMC6034747

[16] Chen DS , Mellman I. Oncology meets immunology: the cancer-immunity cycle. Immunity 2013;39:1–10. 23890059 10.1016/j.immuni.2013.07.012

[17] Elion DL , Jacobson ME, Hicks DJ, Rahman B, Sanchez V, Gonzales-Ericsson PI, . Therapeutically active RIG-I agonist induces immunogenic tumor cell killing in breast cancers. Cancer Res 2018;78:6183–95. 30224377 10.1158/0008-5472.CAN-18-0730

[18] Jacobson ME , Wang-Bishop L, Becker KW, Wilson JT. Delivery of 5′-triphosphate RNA with endosomolytic nanoparticles potently activates RIG-I to improve cancer immunotherapy. Biomater Sci 2019;7:547–59. 30379158 10.1039/c8bm01064a

[19] Johnson LR , Lee DY, Eacret JS, Ye D, June CH, Minn AJ. The immunostimulatory RNA RN7SL1 enables CAR-T cells to enhance autonomous and endogenous immune function. Cell 2021;184:4981–95. 34464586 10.1016/j.cell.2021.08.004 PMC11338632

[20] Hammerich L , Marron TU, Upadhyay R, Svensson-Arvelund J, Dhainaut M, Hussein S, . Systemic clinical tumor regressions and potentiation of PD1 blockade with *in situ* vaccination. Nat Med 2019;25:814–24. 30962585 10.1038/s41591-019-0410-x

[21] Convertine AJ , Benoit DS, Duvall CL, Hoffman AS, Stayton PS. Development of a novel endosomolytic diblock copolymer for siRNA delivery. J Control Release 2009;133:221–9. 18973780 10.1016/j.jconrel.2008.10.004 PMC3110267

[22] Convertine AJ , Diab C, Prieve M, Paschal A, Hoffman AS, Johnson PH, . pH-responsive polymeric micelle carriers for siRNA drugs. Biomacromolecules 2010;11:2904–11. 20886830 10.1021/bm100652w PMC3026907

[23] Garland KM , Sevimli S, Kilchrist KV, Duvall CL, Cook RS, Wilson JT. Microparticle depots for controlled and sustained release of endosomolytic nanoparticles. Cell Mol Bioeng 2019;12:429–42. 31719925 10.1007/s12195-019-00571-6 PMC6816657

[24] Knight FC , Gilchuk P, Kumar A, Becker KW, Sevimli S, Jacobson ME, . Mucosal immunization with a pH-responsive nanoparticle vaccine induces protective CD8(+) lung-resident memory T cells. ACS Nano 2019;13:10939–60. 31553872 10.1021/acsnano.9b00326 PMC6832804

[25] Garland KM , Rosch JC, Carson CS, Wang-Bishop L, Hanna A, Sevimli S, . Pharmacological activation of cGAS for cancer immunotherapy. Front Immunol 2021;12:753472. 34899704 10.3389/fimmu.2021.753472 PMC8662543

[26] Zhang F , Lu Y, Yan S, Xing Q, Tian W. SPRINT: an SNP-free toolkit for identifying RNA editing sites. Bioinformatics 2017;33:3538–48. 29036410 10.1093/bioinformatics/btx473 PMC5870768

[27] Anagnostou V , Bruhm DC, Niknafs N, White JR, Shao XM, Sidhom JW, . Integrative tumor and immune cell multi-omic analyses predict response to immune checkpoint blockade in melanoma. Cell Rep Med 2020;1:100139. 33294860 10.1016/j.xcrm.2020.100139 PMC7691441

[28] Baker AR , Slack FJ. ADAR1 and its implications in cancer development and treatment. Trends Genet 2022;38:821–30. 35459560 10.1016/j.tig.2022.03.013 PMC9283316

[29] Paz-Yaacov N , Bazak L, Buchumenski I, Porath HT, Danan-Gotthold M, Knisbacher BA, . Elevated RNA editing activity is a major contributor to transcriptomic diversity in tumors. Cell Rep 2015;13:267–76. 26440895 10.1016/j.celrep.2015.08.080

[30] Eisenberg E , Levanon EY. A-to-I RNA editing – immune protector and transcriptome diversifier. Nat Rev Genet 2018;19:473–90. 29692414 10.1038/s41576-018-0006-1

[31] Shae D , Becker KW, Christov P, Yun DS, Lytton-Jean AKR, Sevimli S, . Endosomolytic polymersomes increase the activity of cyclic dinucleotide STING agonists to enhance cancer immunotherapy. Nat Nanotechnol 2019;14:269–78. 30664751 10.1038/s41565-018-0342-5 PMC6402974

[32] Li P , Hao Z, Wu J, Ma C, Xu Y, Li J, . Comparative proteomic analysis of polarized human THP-1 and mouse RAW264.7 macrophages. Front Immunol 2021;12:700009. 34267761 10.3389/fimmu.2021.700009 PMC8276023

[33] Jacobson ME , Becker KW, Palmer CR, Pastora LE, Fletcher RB, Collins KA, . Structural optimization of polymeric carriers to enhance the immunostimulatory activity of molecularly defined RIG-I agonists. ACS Cent Sci 2020;6:2008–2022. 33274278 10.1021/acscentsci.0c00568 PMC7706089

[34] Carson CS , Becker KW, Garland KM, Pagendarm HM, Stone PT, Arora K, . A nanovaccine for enhancing cellular immunity via cytosolic co-delivery of antigen and polyIC RNA. J Control Release 2022;345:354–70. 35301055 10.1016/j.jconrel.2022.03.020 PMC9133199

